# Successful surgical treatment for primary cardiac angiosarcoma: a case report

**DOI:** 10.1186/s44215-023-00119-1

**Published:** 2023-11-15

**Authors:** Yasuo Suehiro, Tetsuya Kajiyama, Ayaka Satoh, Hisashi Uemura, Takaya Nakagawa, Hajime Matsue, Hisashi Satoh, Naoto Takase, Hiroshi Doi

**Affiliations:** 1 Department of Cardiovascular Surgery, Higashi Takarazuka Satoh Hospital, 2-1 Nagao-Cho, Takarazuka, Hyogo 665-0873 Japan; 2 Department of Cardiovascular Surgery, Hyogo College of Medicine, Hyogo, Japan; 3 Department of Medical Oncology, Takarazuka City Hospital, Hyogo, Japan; 4 Department of Radiotherapy, Takarazuka City Hospital, Hyogo, Japan

**Keywords:** Primary cardiac angiosarcoma, Surgery, Adjuvant therapy

## Abstract

**Background:**

Primary cardiac angiosarcomas are extremely rare and their prognosis is poor. Surgical resection is the first-line treatment; however, no clear standard of care has been clearly established because of the rarity of these tumors.

**Case presentation:**

A 61-year-old man who had presented with dyspnea on exertion was referred to our hospital. Contrast-enhanced computed tomography revealed massive pericardial effusion and a 40-mm enhanced mass adherent to the anterior wall of the right atrium and involving the right coronary artery. Having diagnosed the mass as a cardiac tumor, we resected the mass under the guidance of epi-cardiac echocardiography guidance, which showed continuity between the tumor and the right atrium, reconstructed the right atrial free wall with a bovine pericardial patch, and performed coronary artery bypass grafting to the right coronary artery using the great saphenous vein. The right atrial wall was resected with adequate tumor-free margin. On the right ventricular side, we resected the right atrial wall 1 cm from the tumor, 2 cm from the atrioventricular groove. Because hemodynamic deterioration occurred after aortic declamping, intra-aortic balloon pumping and veno-arterial extracorporeal membrane oxygenation were instituted. Postoperatively, circulatory support devices were removed safely, and the patient was discharged on the 25th postoperative day. Histopathological examination of the surgical specimens resulted in a diagnosis of angiosarcoma, with positive surgical margins. Chemotherapy and radiotherapy (69 Gy in 30 fractions) were therefore initiated after discharge. To date, the patient has been alive and well with no recurrence of tumor for 4 years and 10 months since surgery.

**Discussion:**

This case study suggests the usefulness of multimodality treatment comprising surgical resection and adjuvant therapy, for cardiac angiosarcoma.

## Background

Primary cardiac angiosarcomas are rare, highly malignant tumors with a poor prognosis. The diagnosis is often delayed because many patients remain asymptomatic. While surgical resection is the gold standard for treatment, adjuvant therapy is also reportedly effective. Additionally, combination therapy is considered to increase survival. We herein report a case of right atrial angiosarcoma successfully treated with surgical resection and postoperative chemoradiation therapy.

## Case presentation

**Fig. 1:**
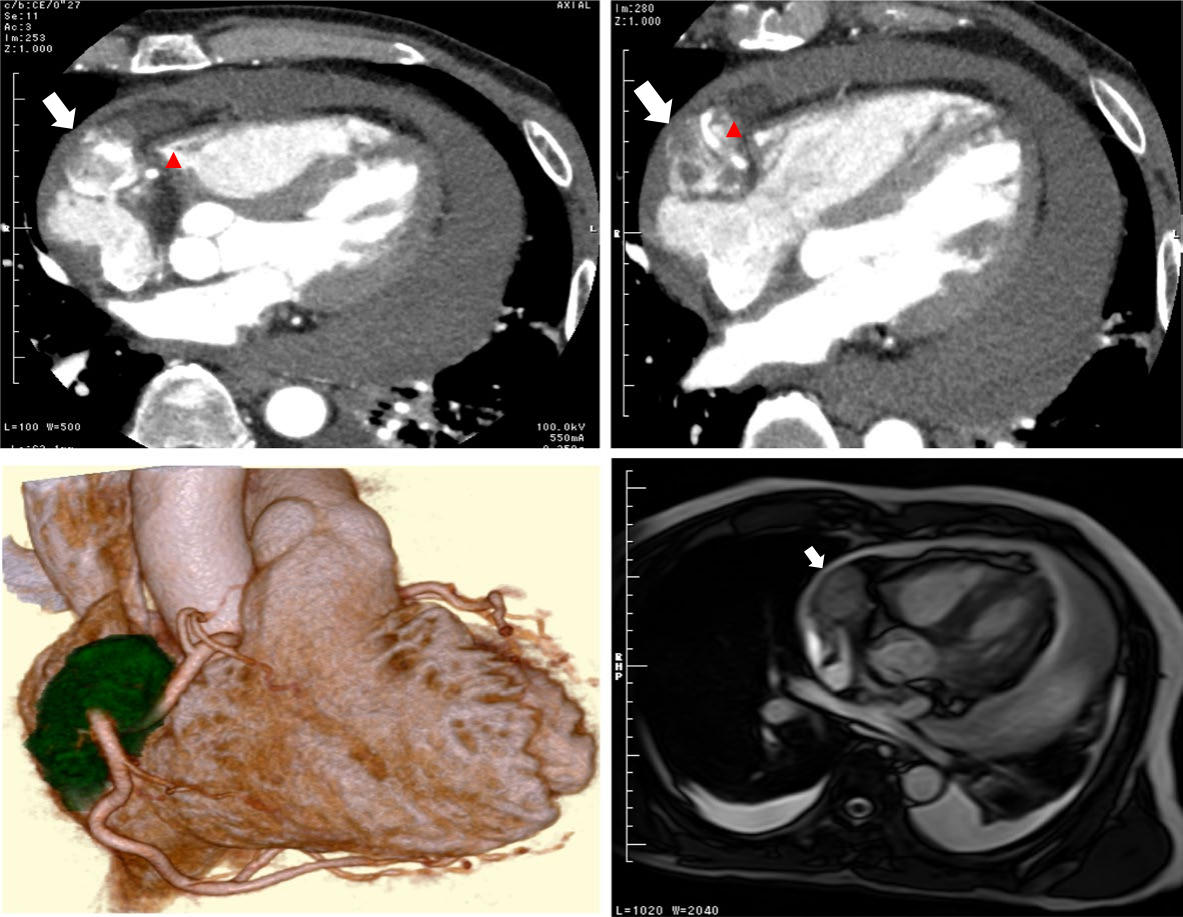
Contrast-enhanced CT images revealing a massive pericardial effusion and a 40-mm enhanced mass (white arrow) adherent to the right atrial wall and involving the RCA (red arrow head). MRI showing the same mass (white arrow), which exhibited an isointense to high signal intensity on T2-weighted images

A 61-year-old man without significant medical history who had presented with dyspnea on exertion was referred to our department for evaluation of a cardiac mass and massive pericardial effusion on computed tomography (CT) images. On admission, physical examinations revealed no abnormal findings, blood pressure of 120/80 mm of mercury, and heart rate of 76 beats per minute. Percutaneous oxygen saturation was 94% at rest in room air, which was slightly decreased compared with normal. Transthoracic echocardiography showed a tumor adjacent to the right atrium and massive pericardial effusion with features of cardiac tamponade. Contrast-enhanced CT revealed a 40-mm hypervascular mass adherent to the anterior wall of the right atrium and involving the right coronary artery (RCA). Magnetic resonance imaging (MRI) also showed this mass at the same location, which exhibited isointense to high signal intensity on T2-weighted images (Fig. 1). No metastasis was confirmed with imaging. Laboratory tests revealed a mildly elevated serum level of C-reactive protein concentration of 3.6 mg/L. Based on these findings, we made a provisional diagnosis of cardiac angiosarcoma arising in the right atrium and scheduled its resection.

**Fig. 2:**
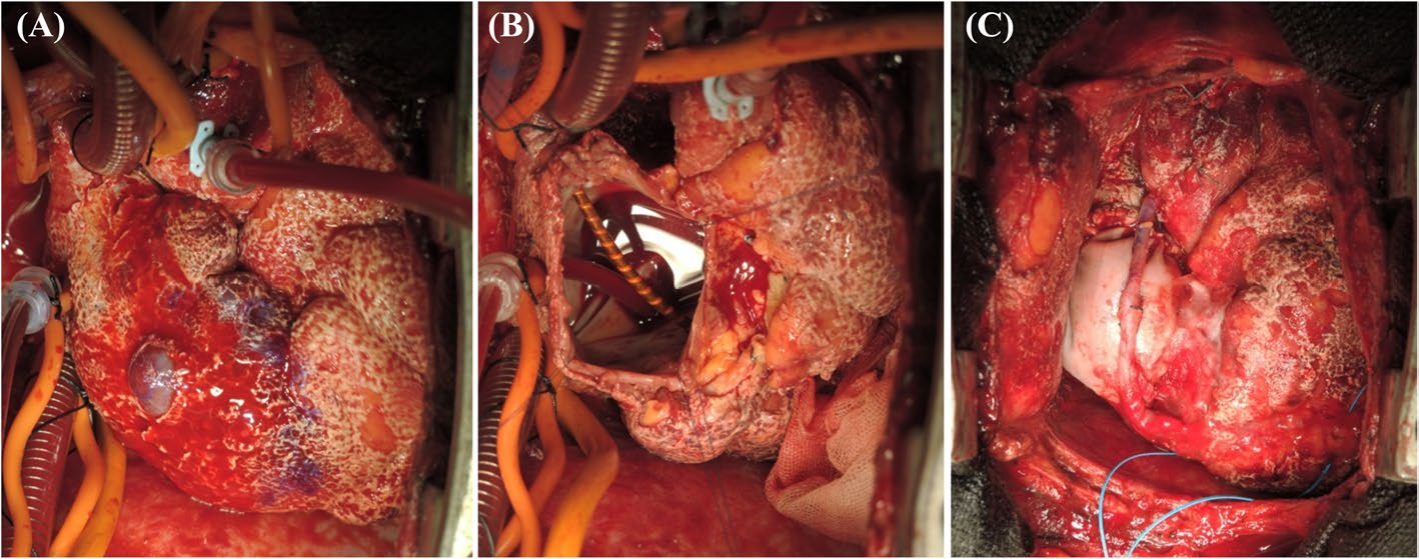
Intraoperative findings. **A** A hard elastic mass protruding anteriorly from the anterior wall of the right atrium. **B** After resection of the tumor together with the right atrial free wall and RCA. **C** After reconstruction of the right atrial free wall with a bovine pericardial patch and coronary artery bypass grafting to the RCA using the great saphenous vein

The procedure was performed through a median sternotomy. A hemorrhagic pericardial effusion and hard mass elastic protruding from the anterior wall of the right atrium were noted intraoperatively. Cardiopulmonary bypass was established after the ascending aorta, superior vena cava, and inferior vena had been cannulated. Cardiac arrest was accomplished with blood cardioplegia. Under the guidance of epi-cardiac echo images of the extent of the tumor and continuity between the tumor and the right atrium, we macroscopically achieved complete resection of the tumor together with the right atrial free wall and RCA. The right atrial wall was resected with adequate tumor-free margins. On the right ventricular side, we resected the right atrial wall 1 cm form the tumor and 2 cm from the atrioventricular groove. We then reconstructed the right atrial free wall with a bovine pericardial patch and performed coronary artery bypass grafting to the RCA using the great saphenous vein (Fig. 2). Because hemodynamic deterioration caused by sacrifice of the right ventricular branches occurred after aortic declamping, intra-aortic balloon pumping and veno-arterial extracorporeal membrane oxygenation were instituted.

**Fig. 3:**
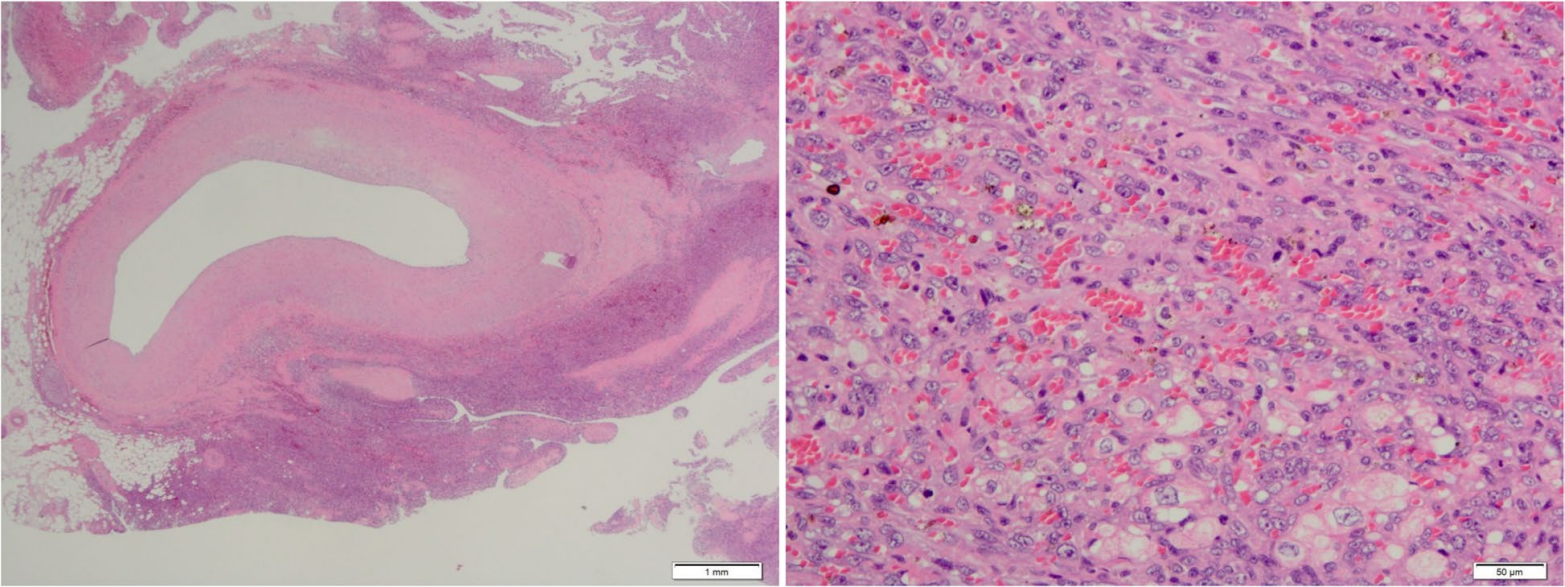
Photomicrographs showing fusiform tumor cells, frequent mitoses, and a vascular neoformation with intramural red blood cells (stain: hematoxylin and eosin; scale bars are included on the figures)

Pathological examination of the resected specimen revealed fusiform tumor cells, frequent mitoses, and a vascular neoformation with intramural red blood cells (Fig. 3), resulting in a diagnosis of primary cardiac angiosarcoma. Resection was incomplete, as shown by positive surgical margins.

Postoperatively, veno-arterial extracorporeal membrane oxygenation and intra-aortic balloon pumping were removed safely on the 2nd and 7th postoperative day, respectively. The patient was discharged on the 25th postoperative day. Chemotherapy for 9 weeks with weekly paclitaxel 80 mg/m^2^ and radiotherapy for 48 days (69 Gy in 30 fractions using intensity modulated radiotherapy) were initiated on the 64th postoperative day. To date, the patient remains alive and well with no recurrence of tumor for 4 years and 10 months postoperatively.

## Discussion

Primary cardiac tumors are extremely rare, with an autopsy incidence ranging from 0.001–0.28%. Approximately 25% of cardiac tumors are malignant, cardiac angiosarcomas are the commonest of these, accounting for 30% [1, 2]. Seventy percent of cardiac angiosarcomas arise in the right atrium [3], and metastases are present at the time of diagnosis in approximately 66–89% of cases [4]. Angiosarcomas have a poor prognosis, with a mean survival of 6 months. Furthermore, even in surgically-treated cases, survival time after surgery is likely to be within 10 months [1]. Because many patients remain asymptomatic until the tumor reaches a certain size, diagnosis is often delayed.

Imaging tests, such as echocardiography, CT, MRI, and fluorodeoxyglucose-positron emission tomography, are helpful for the detection and accurate diagnosis of cardiac tumors. Such imaging can provide us information about the presence of both a tumor and metastases, as well as the relationship between the tumor and the surrounding structures. Contrast-enhanced CT shows heterogeneous enhancement and MRI shows heterogeneous T2-weightned signal intensity patterns, as in the present case. Histopathology is necessary for a definitive diagnosis, pericardial fluid cytology having a low diagnostic yield [4]. Because needle biopsy carries risks of bleeding and inducing metastatic spread, it may be preferable to obtaining a specimen surgically.

Resection is the standard first-line treatment for cardiac angiosarcomas. Hamidi et al. reported that the median survival of those who undergo surgery is 12 months, whereas survival is 1 month for those who do not [5]. According to Bakaeen et al., complete resection of these tumors achieves better overall survival than does incomplete resection [6]. When the tumor is large, early surgical intervention is recommended because of concerns regarding intra-cardiac obstruction by the tumor, and occasionally incarceration, of the tumor.

In the present case, after the tumor had been confirmed by epi-cardiac echo intraoperatively to have continuity with the right atrium, we macroscopically achieved complete resection of the right atrial free wall along with the tumor. However, histopathological examination revealed positive margins on the side of the right ventricle. If we had tried to achieve adequate tumor-free margins, reconstruction of the right ventricle and tricuspid valve would have been necessary. When deciding on the extent of resection, surgeons must consider the feasibility and difficulty of reconstructing cardiac structures and the procedure’s disadvantages, including prolonged cardiac arrest time.

Chemotherapy and radiotherapy are the most commonly used forms of adjuvant therapy for angiosarcomas; however, their benefits are controversial because of the rarity of these tumors. Luk, A. et al. reported the efficacy of postoperative chemotherapy, radiotherapy, or a combination of these [7]. Furthermore, patients treated by complete resection alone characteristically rapidly develop metastases postoperatively [8], whereas some who undergo complete resection and postoperative chemoradiation therapy do not develop recurrences thereafter [9, 10], even in cases of negative surgical margins.

The traditional chemotherapy drugs for angiosarcoma are anthracycline, ifosfamide, and taxanes (paclitaxel and docetaxel). Recently, taxanes have displayed good efficacy as adjuvant chemotherapy [11], and in particular, weekly paclitaxel has demonstrated clinical benefit [12]. Regarding the radiotherapy, National Comprehensive Cancer Network guideline recommends over 70 Gy of external beam radiotherapy as definitive treatment of unresectable soft tissue sarcomas [13]. The safety and efficacy of this treatment regimen of intensity modulated radiotherapy using the simultaneous integrated boost technique for sarcomas have been reported [14, 15]. Besides, combination of weekly paclitaxel and radiotherapy is reportedly more effective for cardiac angiosarcoma [16], as described in the present case. We believe that postoperative adjuvant therapy is warranted regardless of whether complete resection is achieved. Heart transplantation is a possible therapeutic option, but too few patients have undergone this treatment to allow assessment of its survival benefit.

## Conclusion

We presented a case of right atrial angiosarcoma successfully treated with surgical resection and postoperative chemoradiation therapy. Achieving complete resection is the most important prognostic factor. We believe that multimodality approaches comprising surgery and adjuvant therapy are crucial for improving overall survival.

## Data Availability

There are no additional data to disclose.

## Abbreviations

CT: Computed tomography
RCA: Right coronary artery
MRI: Magnetic resonance imaging
