# Correction: Successful surgical treatment for primary cardiac angiosarcoma: a case report

**DOI:** 10.1186/s44215-024-00127-9

**Published:** 2024-02-26

**Authors:** Yasuo Suehiro, Tetsuya Kajiyama, Ayaka Satoh, Hisashi Uemura, Takaya Nakagawa, Hajime Matsue, Hisashi Satoh, Naoto Takase, Hiroshi Doi

**Affiliations:** 1 Department of Cardiovascular Surgery, Higashi Takarazuka Satoh Hospital, 2‑1 Nagao‑Cho, Takarazuka, Hyogo 665‑0873 Japan; 2 Department of Cardiovascular Surgery, Hyogo College of Medicine, Hyogo, Japan; 3 Department of Medical Oncology, Takarazuka City Hospital, Hyogo, Japan; 4 Department of Radiotherapy, Takarazuka City Hospital, Hyogo, Japan

**Correction: Gen Thor Cardiovasc Surg Cases 2, 94 (2023)**

**https://doi.org/10.1186/s44215-023-00119-1**

Following publication of the original article [1], we have been notified that two authors had to be added to the published article.

It is now:

Yasuo Suehiro1*, Tetsuya Kajiyama2, Ayaka Satoh1, Hisashi Uemura1, Takaya Nakagawa1, Hajime Matsue1 and Hisashi Satoh1

It should be as per below:

Yasuo Suehiro1*, Tetsuya Kajiyama2, Ayaka Satoh1, Hisashi Uemura1, Takaya Nakagawa1, Hajime Matsue1, Hisashi Satoh1, Naoto Takase 3 and Hiroshi Doi 4

The original article was updated.
